# Supplementary material for: Realistic mossy fiber input patterns to unipolar brush cells evoke a continuum of temporal responses comprised of components mediated by different glutamate receptors
Source: bioRxiv. 2024 Sep 17:2024.09.17.613480. Preprint. [Version 1] doi: 10.1101/2024.09.17.613480 (PMC11429827; doi:10.1101/2024.09.17.613480)
Supplement: Supplementary file 1 [file NIHPP2024.09.17.613480v1-supplement-1.pdf]

bioRxiv preprint doi: https://doi.org/10.1101/2024.09.17.613480; this version posted September 17, 2024. The copyright holder for this preprint (which was not certified by peer review) is the author/funder, who has granted bioRxiv a license to display the preprint in perpetuity. It is made available under aCC-BY 4.0 International license.

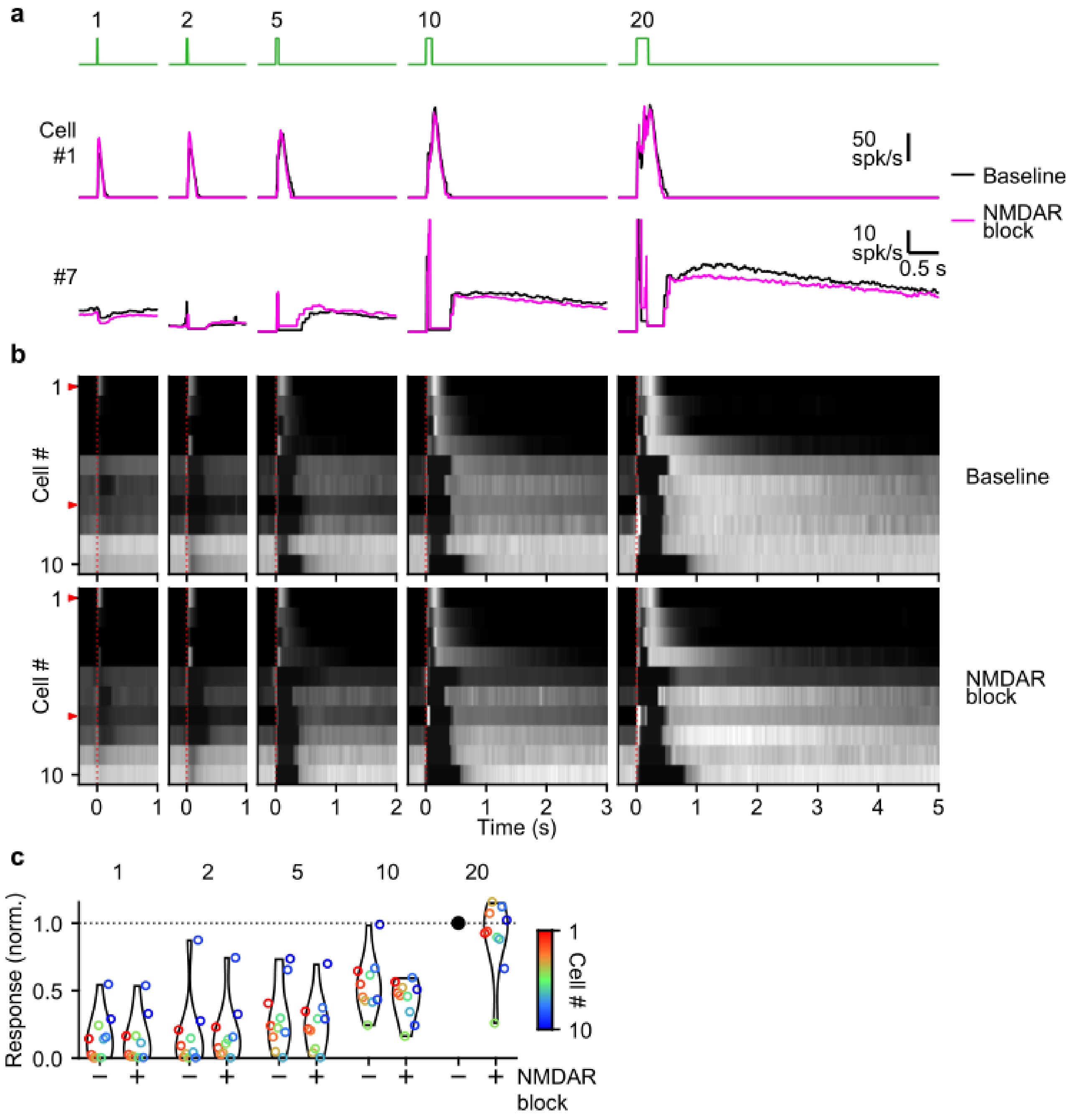

Figure 3 - Supplementary Figure 1: NMDA receptors do not significantly contribute to burst responses

a. Instantaneous firing rates of two representative UBCs in response to 100 spk/s bursts comprised of 1-20 stimuli (stimulus indicated above in green). Responses shown for baseline conditions (black), and after addition of an antagonist of NMDA receptors (pink). Cell numbers refer to the index in the summary plot **b**.

bioRxiv preprint doi: https://doi.org/10.1101/2024.09.17.613480; this version posted September 17, 2024. The copyright holder for this preprint (which was not certified by peer review) is the author/funder, who has granted bioRxiv a license to display the preprint in perpetuity. It is made available under aCC-BY 4.0 International license.

b. Heat maps showing the normalized instantaneous firing rates for all UBCs in response to 100 spk/s burst comprised of 1-20 stimuli (*columns*), for baseline and after addition of an NMDA receptor antagonist (*rows*). Responses were normalized per cell to the peak firing rate in the baseline response to the 20 stimuli at 100 spk/s burst. Cells sorted by their response to the baseline 20 stimuli at 100 spk/s input, either by the half-width of the increase in firing (cell #1-8) or by pause duration (cell #9-10). Time indicates seconds since start of MF stimulation (indicated by dotted red line). Red arrows indicate representative UBCs shown in **a.**
c. Violin plots of the number of spikes after each burst under baseline conditions (-) and after blocking NMDAR (+), normalized to the number of spikes after 20 stimuli at 100 spk/s under baseline conditions. Markers indicate individual UBCs color coded by the cell index in **b**.

bioRxiv preprint doi: https://doi.org/10.1101/2024.09.17.613480; this version posted September 17, 2024. The copyright holder for this preprint (which was not certified by peer review) is the author/funder, who has granted bioRxiv a license to display the preprint in perpetuity. It is made available under aCC-BY 4.0 International license.

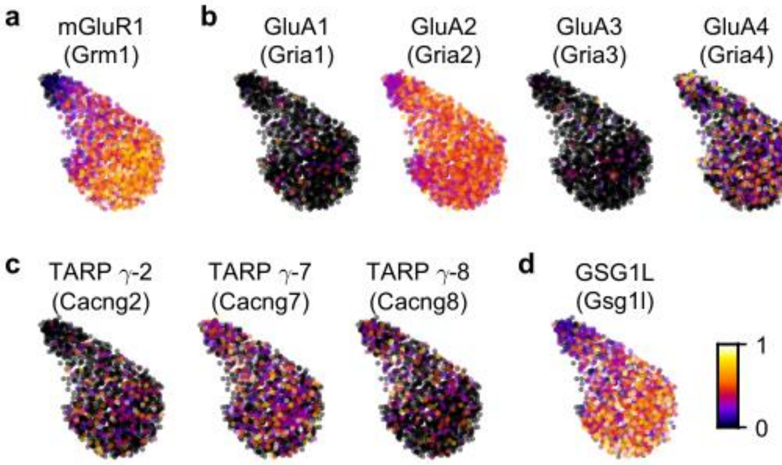

Figure 3 - Supplementary Figure 2: AMPAR auxiliary subunits are expressed differentially in the UBC population

a. UMAP embedding of normalized gene expression in the UBC population for mGluR1.

b. Same as in **a** but showing AMPAR subunits GluA1-4.

c. Same as in **a** but showing auxiliary subunits TARP γ-2, γ-7, and γ-8.

d. Same as in **a** but showing auxiliary subunit GSG1L.

bioRxiv preprint doi: https://doi.org/10.1101/2024.09.17.613480; this version posted September 17, 2024. The copyright holder for this preprint (which was not certified by peer review) is the author/funder, who has granted bioRxiv a license to display the preprint in perpetuity. It is made available under aCC-BY 4.0 International license.

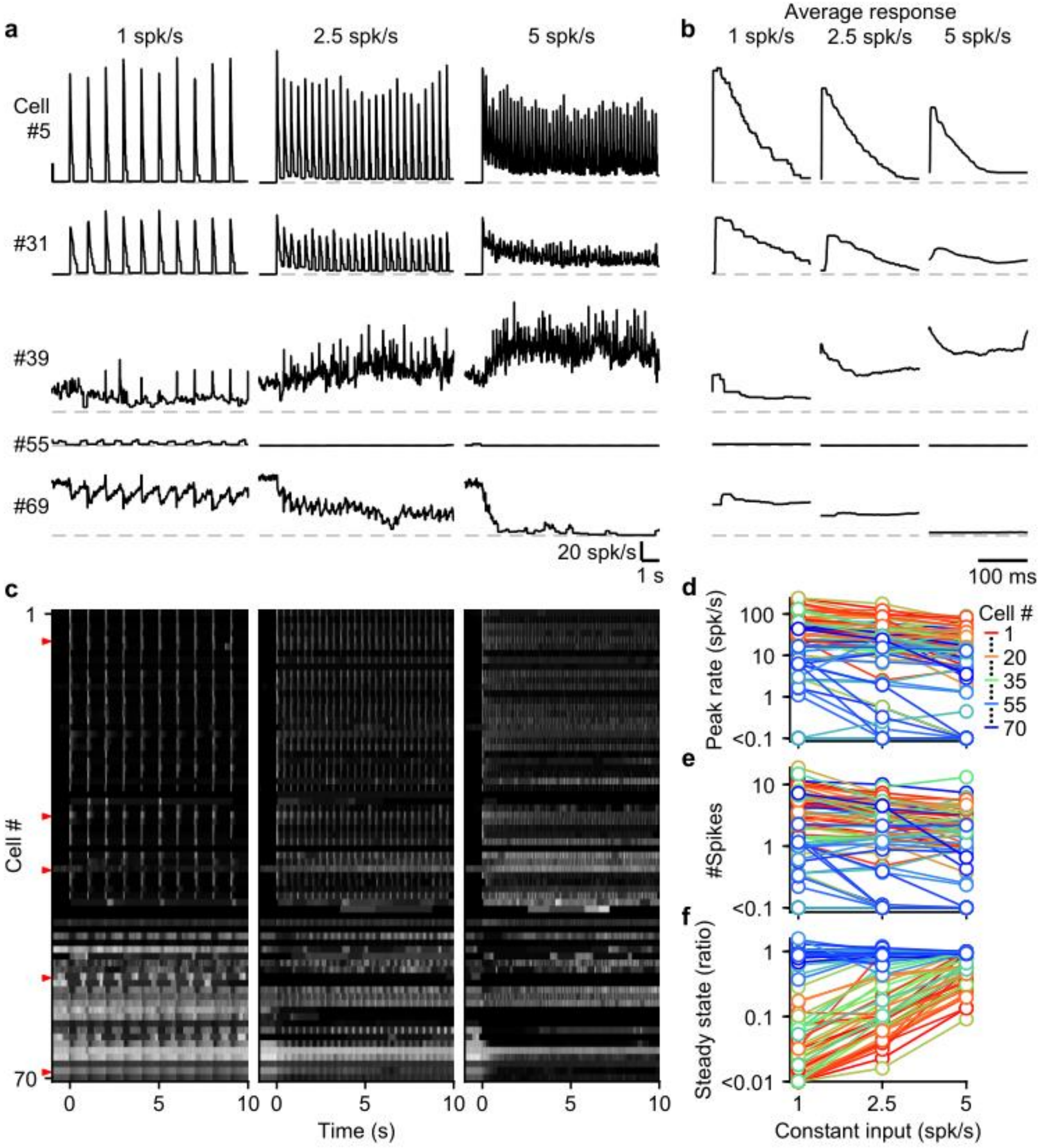

Figure 4 - Supplementary Figure 1: UBC responses to sustained 1 to 5 spk/s MF input.

a. Instantaneous firing rates of five representative UBCs in response to sustained MF input at three different rates (1, 2.5, and 5 spk/s). Dashed gray lines indicate 0 spk/s; cell numbers refer to the index in the summary plot **c**.

b. As in **a** but for the average response of all individual inputs excluding the first 5.

bioRxiv preprint doi: https://doi.org/10.1101/2024.09.17.613480; this version posted September 17, 2024. The copyright holder for this preprint (which was not certified by peer review) is the author/funder, who has granted bioRxiv a license to display the preprint in perpetuity. It is made available under aCC-BY 4.0 International license.

c. Heat maps showing the normalized instantaneous firing rates for all UBCs in response to sustained input at 1, 2.5, and 5 spk/s. Responses normalized per cell by their peak firing rate. Cells sorted by their response to 20 stimuli at 100 spk/s bursts as in **Fig. 2b**. Time indicates seconds since start of sustained MF input. Red arrows indicate representative UBCs shown in **a**,**b**.

d. Summary plot showing the peak rate of the average response (as in **b)** for all UBCs color coded to correspond to the cell index in **c**.

e. As in **d** but for the average number of spikes fired.

f. As in **d** but for the ratio between the average steady state firing rate (200 ms after stimuli) and the peak firing rate shown in **d**.

bioRxiv preprint doi: https://doi.org/10.1101/2024.09.17.613480; this version posted September 17, 2024. The copyright holder for this preprint (which was not certified by peer review) is the author/funder, who has granted bioRxiv a license to display the preprint in perpetuity. It is made available under aCC-BY 4.0 International license.

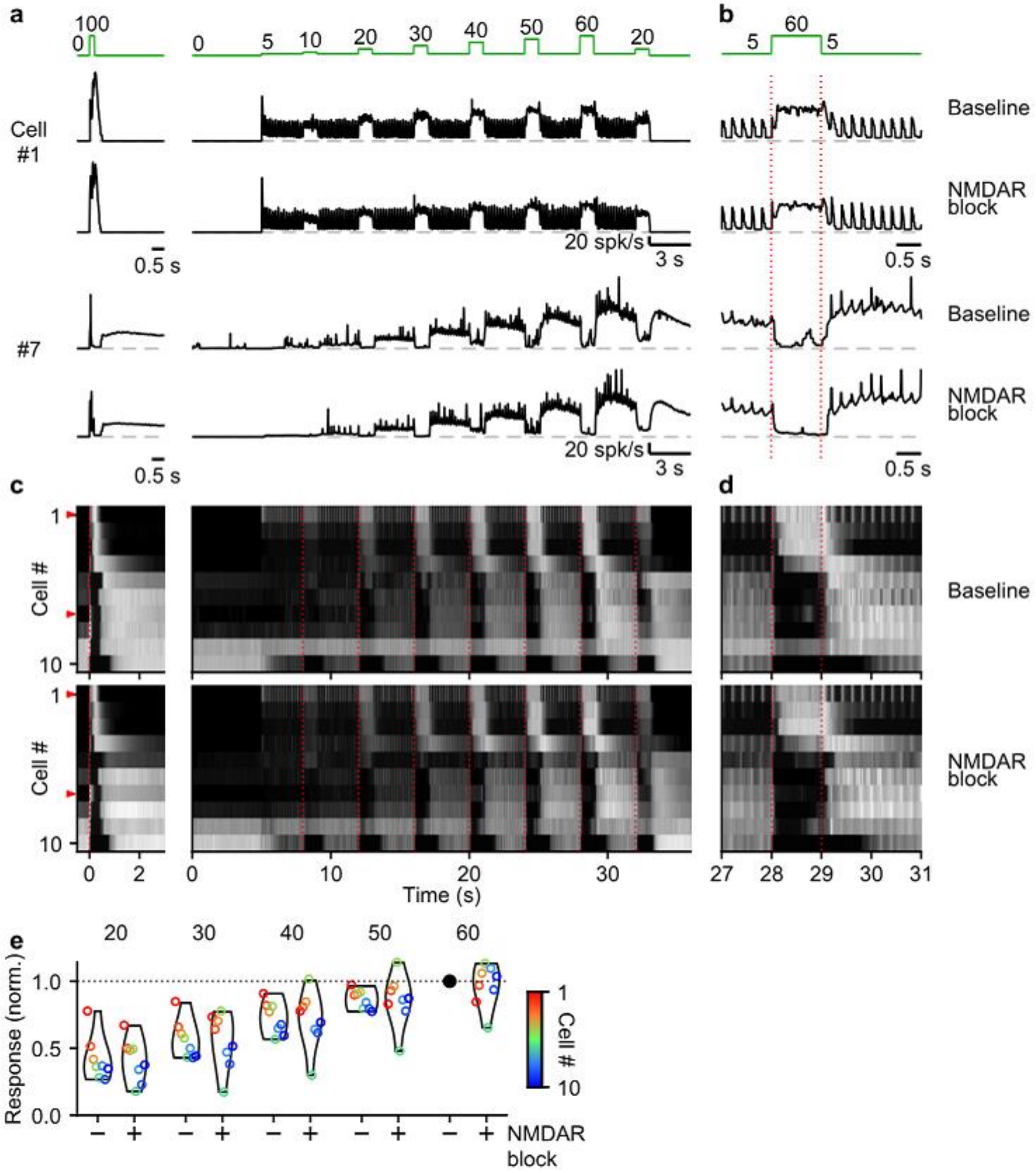

Figure 5 - Supplementary Figure 1: NMDA receptors do not significantly contribute to responses to smooth pursuit-like input

a. Instantaneous firing rates of two representative UBCs in response to 20 stimuli at 100 spk/s burst (*left*), and smooth pursuit-like MF input (*right*) as indicated by the traces at the top (green).

bioRxiv preprint doi: https://doi.org/10.1101/2024.09.17.613480; this version posted September 17, 2024. The copyright holder for this preprint (which was not certified by peer review) is the author/funder, who has granted bioRxiv a license to display the preprint in perpetuity. It is made available under aCC-BY 4.0 International license.

Responses shown under baseline conditions and after addition of an antagonist of NMDA receptors. Dashed gray lines indicate 0 spk/s; cell numbers refer to the index in the summary plot **c**.

b. The same as in **a** but on an expanded timescale, displaying only the 60 spk/s step. Dotted red lines indicate onset and offset of the 60 spk/s step.

c. Heat maps showing the normalized instantaneous firing rates for all UBCs in response 20 stimuli at 100 spk/s bursts (*left*) and smooth pursuit-like MF input (*right*), for baseline and after addition of NMDA receptor antagonist (*rows*). Responses normalized to the peak firing rate during baseline conditions separately for burst and smooth pursuit-like MF input. Cells sorted by their response to the baseline 20 stimuli at 100 spk/s input, either by the half-width of the increase in firing (cell #1-8) or by the pause duration (cell #9-10). Dotted red lines indicate the start of the step changes in input rate, red arrows indicate representative UBCs shown in **a,b**.

d. The same as in **c** but on an expanded timescale, displaying only the 60 spk/s step.

e. Violin plots of the number of spikes in the 1 s period during the step and the 3 s period after under baseline conditions (-) and after blocking NMDAR (+), normalized to the number of spikes associated with the step to 60 spk/s under baseline conditions. Markers indicate individual UBCs color coded by the cell index in **c**.

bioRxiv preprint doi: https://doi.org/10.1101/2024.09.17.613480; this version posted September 17, 2024. The copyright holder for this preprint (which was not certified by peer review) is the author/funder, who has granted bioRxiv a license to display the preprint in perpetuity. It is made available under aCC-BY 4.0 International license.

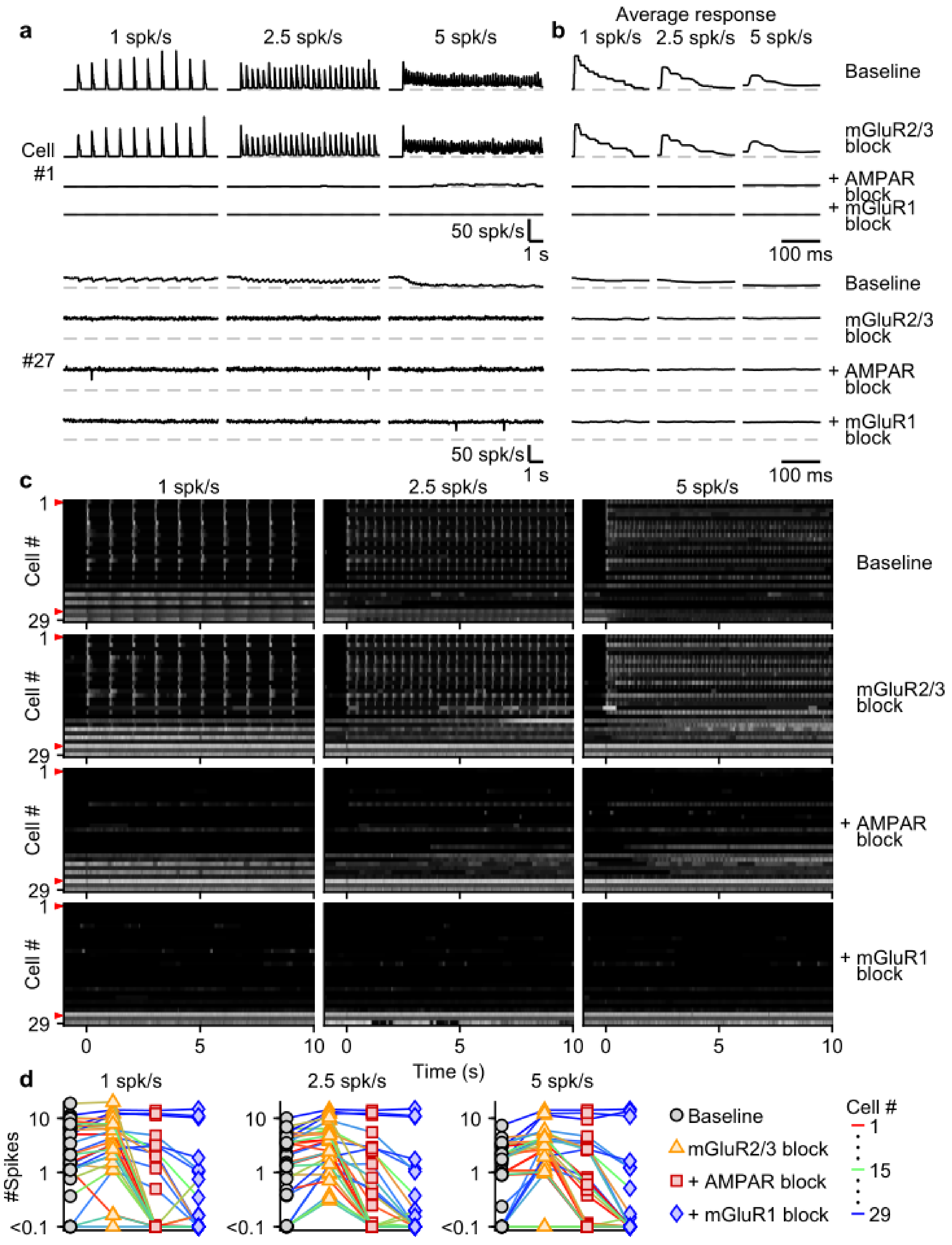

bioRxiv preprint doi: https://doi.org/10.1101/2024.09.17.613480; this version posted September 17, 2024. The copyright holder for this preprint (which was not certified by peer review) is the author/funder, who has granted bioRxiv a license to display the preprint in perpetuity. It is made available under aCC-BY 4.0 International license.

Figure 5 - Supplementary Figure 2: Contribution of different glutamate receptors to UBC responses evoked by sustained 1 to 5 Hz MF stimulation.

a. Instantaneous firing rates of two representative UBCs in response to sustained MF input at three different rates (1, 2.5, and 5 spk/s). Responses shown under baseline conditions and after successive addition of antagonists of mGluR2/3, AMPAR, and mGluR1. Dashed gray lines indicate 0 spk/s; cell numbers refer to the index in the summary plot **c**.

b. As in **a** but for the average response of all individual inputs excluding the first 5.

c. Heat maps showing the normalized instantaneous firing rates for all UBCs in response to sustained input at 1, 2.5, and 5 spk/s (*columns*), for baseline and after addition of glutamate receptor antagonists (*rows*). Responses normalized per cell to the peak firing rate after application of the mGluR2/3 antagonist. Cells sorted by their response to 20 stimuli at 100 spk/s bursts under baseline conditions as in **Fig. 3d** (with exception of **Fig. 3** cell #20 and #24).

d. Summary plot showing the number of spikes fired in the average response to 1, 2.5, and 5 spk/s sustained input (as in **b**) for all UBCs color coded to correspond to the cell index in **c**. Successive different markers indicate baseline and the different glutamate antagonists.
